# From Emergency Department to Operating Room: The Role of Early Prehabilitation and Perioperative Care in Emergency Laparotomy: A Scoping Review and Practical Proposal

**DOI:** 10.3390/jcm14196922

**Published:** 2025-09-30

**Authors:** Francisco Javier García-Sánchez, Fernando Roque-Rojas, Natalia Mudarra-García

**Affiliations:** 1 Emergency Room Service, Surgical Prehabilitation Unit, Hospital Universitario Infanta Cristina, Instituto de Investigación Sanitaria Hospital Puerta de Hierro Segovia Arana (IDIPHISA), 28981 Madrid, Spain; fernando.roque@salud.madrid.org; 2 Medical Department, Faculty of Medicine, University Complutense of Madrid, 28040 Madrid, Spain; 3 Instituto Ramón y Cajal de Investigación Sanitaria (IRYCIS), 28034 Madrid, Spain; nmudarra@enf.ucm.es; 4 Nursing Department, Faculty of Nurse, Phisiotherapy and Podology, University Complutense of Madrid, 28040 Madrid, Spain

**Keywords:** emergency general surgery, prehabilitation, frailty, sarcopenia, gastrointestinal surgery, Clinical Frailty Scale, SARC-F, ERAS

## Abstract

Background: Emergency laparotomy (EL) carries high morbidity and mortality relative to elective abdominal surgery. While Enhanced Recovery After Surgery (ERAS) principles improve outcomes in elective care, their translation to emergencies is inconsistent. The emergency department (ED) provides a window for rapid risk stratification and pre-optimization, provided that interventions do not delay definitive surgery. Methods: We conducted a PRISMA-ScR–conformant scoping review to map ED-initiated, ERAS-aligned strategies for EL. PubMed, Scopus, and Cochrane were searched in February 2025. Eligible sources comprised ERAS guidelines, systematic reviews, cohort studies, consensus statements, and programmatic reports. Evidence was charted across five a priori domains: (i) ERAS standards, (ii) comparative effectiveness, (iii) ED-feasible pre-optimization, (iv) risk stratification (Emergency Surgery Score [ESS], frailty, sarcopenia), and (v) oncological emergencies. Results: Thirty-four sources met inclusion. ERAS guidelines codify rapid assessment, multimodal intraoperative care, and early postoperative rehabilitation under a strict no-delay rule. Meta-analysis and cohort data suggest ERAS-aligned pathways reduce complications and length of stay, though heterogeneity persists. ED-feasible measures include multimodal analgesia, goal-directed fluids, early safe nutrition, respiratory preparation, and anemia/micronutrient optimization (IV iron, vitamin B12, folate, vitamin D). Sarcopenia, frailty, and ESS consistently predicted adverse outcomes, supporting targeted bundle activation. Evidence from oncological emergencies indicates feasibility under no-delay governance. Conclusions: A minimal, ED-initiated, ERAS-aligned bundle is feasible, guideline-concordant, and may shorten hospitalization and reduce complications in EL. We propose a practical framework that links rapid risk stratification, opportunistic pre-optimization, and explicit continuity into intra- and postoperative care; future studies should test fidelity, costs, and outcome impact in pragmatic emergency pathways.

## 1. Introduction

Emergency laparotomy (EL) remains one of the highest-risk procedures in general surgery, with consistently reported mortality rates between 10 and 20% and morbidity exceeding 40% in many cohorts [1]. These outcomes stand in sharp contrast to comparable elective procedures, where perioperative pathways and prehabilitation programs have significantly improved recovery, reduced complications, and shortened hospital stays [2]. Despite its frequency and impact, EL is often characterized by variability in perioperative care delivery, resource allocation, and postoperative trajectories, especially in elderly and frail populations [3].

International societies have increasingly emphasized that structured perioperative pathways—most notably, the Enhanced Recovery After Surgery (ERAS) approach—should not be confined to elective settings but should also be adapted for urgent procedures as well [4,5,6]. ERAS principles, including early nutrition, goal-directed fluid therapy, multimodal analgesia, pulmonary optimization, and early mobilization, have become the standard of care in elective surgery. Their translation into emergency contexts, however, requires modification to accommodate compressed timelines, diagnostic uncertainty, and the imperative not to delay definitive source control [7,8].

Meta-analysis and comparative cohort studies in emergency abdominal surgery suggest that ERAS-aligned pathways are both feasible and associated with beneficial outcomes, such as reduced length of stay, fewer postoperative complications, and improved functional recovery [7,8]. However, implementation remains inconsistent, and the “window of opportunity” for pre-optimization is often underused in emergency departments (EDs), where patients may spend hours awaiting diagnostic clarification, resuscitation, or operating room (OR) availability [5,9].

A targeted approach is essential. Sarcopenia and frailty are now well-recognized as strong, independent predictors of postoperative complications, prolonged length of stay, institutionalization, and mortality in emergency surgical populations [10,11,12]. Sarcopenia may already be present before surgery or develop acutely in the perioperative course (“acute sarcopenia”), compounding vulnerability [11]. Frailty assessment tools and rapid scoring systems, such as the Emergency Surgery Score (ESS), provide clinicians with a pragmatic way to stratify risk and allocate resources in busy emergency department (ED) settings [13].

Moreover, oncological emergencies, such as obstructive colorectal cancer, present a frequent and particularly high-risk subset where the principles of ERAS can and should be adapted, provided that no-delay governance and structured handover into peri-operative orders are guaranteed [14].

Objective. The aim of this scoping review is to synthesize current evidence on emergency department (ED)-initiated peri-operative care aligned with enhanced recovery after surgery (ERAS) for emergency laparotomy (EL), including standards and guidelines, comparative effectiveness, feasible pre-optimization components, targeting strategies using frailty, sarcopenia, and Emergency Surgery Score (ESS), and contextual evidence from oncology emergencies. From this synthesis, we propose a minimal, pragmatic, ED-initiated bundle that is feasible, safe, and transferable to clinical practice.

Definitions used in this review. No-delay governance denotes a safety rule whereby any ED-initiated optimization must not postpone source control or time-to-operating room (OR); actions are opportunistic and abandoned if they risk delay. Bundle fidelity denotes adherence to predefined components (analgesia/fluids, nutrition, respiratory, patient blood management) and their continuity across ED → OR → ward.

Unlike previous systematic reviews focused on outcome estimates in selected domains, this scoping review uniquely maps ED-initiated, ERAS-aligned pre-optimization and translates feasibility, targeting, and implementation into a practical, tiered pathway for real-world emergency care.

## 2. Materials and Methods

### 2.1. Rationale and Design

A scoping review methodology was selected because the field of perioperative prehabilitation in the context of emergency laparotomy (EL) is heterogeneous, rapidly evolving, and insufficiently mapped for formal meta-analysis. Scoping reviews enable the identification of concepts, evidence gaps, and implementation opportunities across diverse study designs. The methodology followed Arksey and O’Malley’s framework, which was expanded by Levac et al., and conformed to the PRISMA-ScR checklist to enhance transparency and reproducibility.

### 2.2. Research Questions

The review was guided by the following questions:

1. What ERAS standards and guidelines currently exist for emergency laparotomy?
2. What evidence supports the feasibility and effectiveness of ERAS-aligned interventions in emergency surgery?
3. Which components of pre-optimization are feasible to initiate in the emergency department (ED) without delaying surgery?
4. Which tools (e.g., ESS, sarcopenia, frailty indices) are available for rapid risk stratification in ED settings?
5. What evidence exists regarding the contextual application in oncological emergencies, particularly in obstructive or complicated colorectal cancer?

### 2.3. Eligibility Criteria (PCC Framework)

- Population: Adults (≥18 years) undergoing emergency gastrointestinal or hepatopancreatobiliary surgery, with an emphasis on EL.
- Concept: Interventions or care bundles aligned with ERAS and perioperative optimization, including nutrition, respiratory training, mobilization, delirium prevention, anemia/micronutrient correction, fluid and analgesia strategies, and implementation science. Also included were risk stratification tools (ESS, frailty, sarcopenia).
- Context: EDs, acute surgical admission units, and perioperative emergency pathways (including oncological emergencies).

### 2.4. Search Details and Reviewer Agreement

We searched PubMed/MEDLINE, Scopus and Cochrane (inception to February 2025, the final pre-submission update). An example MEDLINE strategy was: (emergency laparotomy OR emergency abdominal surgery OR emergency general surgery) AND (enhanced recovery OR ERAS OR prehabilitation OR pre-optimization OR preoptimization) AND (frailty OR sarcopenia OR “Emergency Surgery Score” OR ESS OR “patient blood management” OR anemia OR iron). Humans; adults. Strategies were adapted for Scopus and Cochrane. We included grey literature limited to society guidelines/consensus with explicit peri-operative recommendations. Two reviewers screened titles/abstracts and full texts independently after a calibration exercise (piloted 50 records) using a piloted charting form. Disagreements were resolved by discussion; a senior reviewer adjudicated unresolved cases. Data extraction was duplicated for a random 20% sample and reconciled to ensure inter-rater consistency.

### 2.5. Study Selection

**Figure 1:**
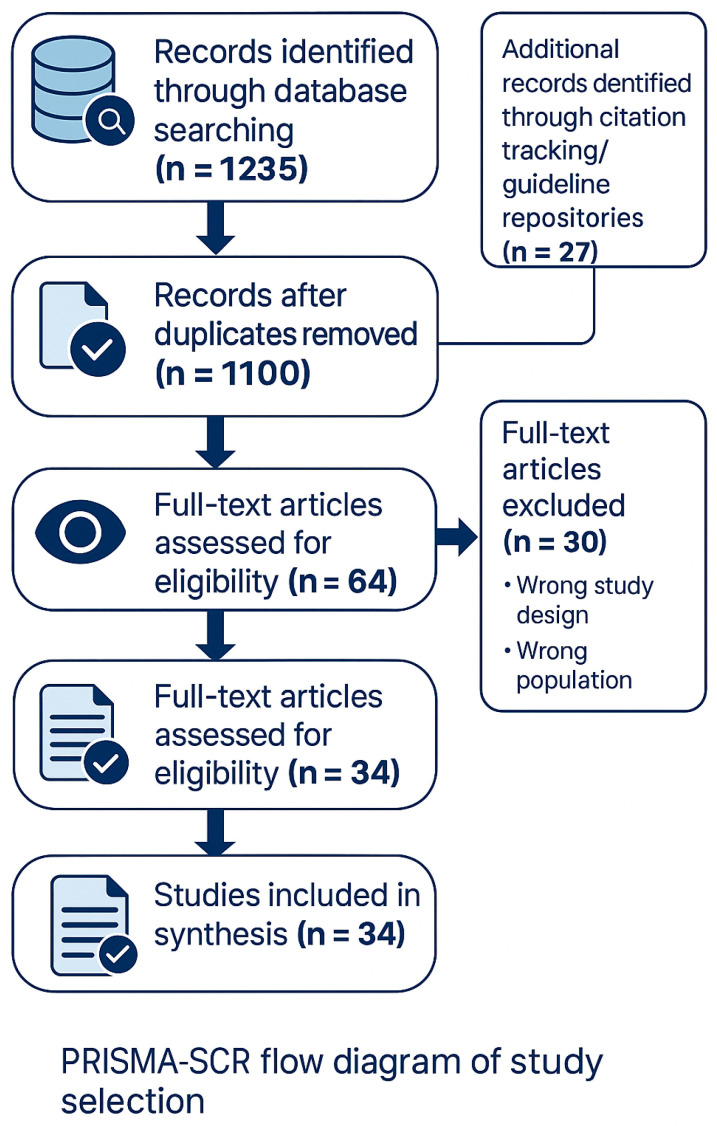
PRISMA-ScR flow diagram of study selection (n = 34). Records identified through database searching (n = 1235) and citation tracking/guideline repositories (n = 27). After de-duplication, records screened (n = 1100); full-text articles assessed for eligibility (n = 64); full-text articles excluded with reasons (n = 30); studies included in the synthesis (n = 34).

Two reviewers independently screened titles and abstracts and assessed full texts. Disagreements were resolved by consensus. A PRISMA-ScR flow diagram will be provided in Figure 1.

### 2.6. Data Extraction and Charting

Data were extracted into a structured template capturing study design, setting, population, intervention domains (nutrition, respiratory, mobilization, delirium prevention, anemia/micronutrients, analgesia/fluids, implementation), and outcomes (mortality, morbidity, length of stay (LOS), discharge destination, functional recovery, transfusion, feasibility). Risk stratification tools were extracted separately.

### 2.7. Synthesis of Results

Given the heterogeneity, a narrative synthesis was undertaken. Evidence was grouped into five domains: (i) ERAS standards; (ii) comparative outcomes; (iii) ED-initiated pre-optimization; (iv) targeting and risk stratification; and (v) oncological emergencies. Gaps and implementation implications were identified.

Beyond ERAS and ED pre-optimization literature, we explicitly charted emergent domains relevant to ED triage and handover—CT-based body composition, preoperative immune biomarkers, delirium risk prediction, peri-operative cardiovascular guidance, and dyslipidemia to capture operationally actionable evidence. These domains included sarcopenia/myosteatosis in EL, biomarker-augmented risk models (suPAR: soluble urokinase plasminogen activator receptor, IL-6, TNF-α), validated delirium prediction (PIPRA: Pre-Interventional Preventive Risk Assessment), ESC non-cardiac surgery recommendations, and dyslipidemia outcome links.

Methodological appraisal (light-touch). In line with contemporary scoping practice, we appraised methodological signals by study type using brief, fit-for-purpose checklists: selected AMSTAR-2 items for systematic reviews, ROBINS-I signal domains for non-randomized cohort studies, and AGREE-II signal items for guidelines. Two reviewers appraised independently; disagreements were resolved by consensus with senior adjudication when needed. Judgments are reported in three tiers (low concerns/some concerns/serious concerns) and summarized in Supplementary Table S2 (“Methodological Appraisal Signals by Study”).

### 2.8. Patient Selection and Decision Rules for ED Activation

- Full bundle if ESS ≥ 7 or Clinical Frailty Scale (CFS) ≥ 5 or documented sarcopenia (rectus femoris ultrasound below age/sex thresholds), and expected time-to-OR ≥ 3 h.
- Partial bundle (analgesia/fluids ± respiratory coaching) if ESS 4–6 or CFS 4; defer nutrition if aspiration risk or bowel obstruction is suspected.
- Contraindications: imminent transfer to OR (<2 h); sepsis-induced hemodynamic instability (defer IV iron); high aspiration risk/bowel obstruction (no ONS); severe hypoxemia or agitation precluding coaching.
- Concurrency rules: when ≥2 high-risk arrivals compete, prioritize by (a) time-to-OR and (b) respiratory risk (e.g., SpO…OR and (b) respiratory risk (e.g., SpO_2_ < 92%, COPD) for early physiotherapy; nutrition tasks can shift to ward handover.

## 3. Results

### 3.1. Evidence Base

The synthesis incorporated 34 sources: ERAS Society guidelines for EL (Part 1 and Part 2) [5,6]; a systematic review of emergency laparotomy pathways [4]; a meta-analysis and cohort evidence of ERAS in emergencies [7,8,15]; best-practice and consensus documents on pre-optimization and peri-operative anemia/PBM [9,16,17,18,19]; evidence on sarcopenia, frailty, ESS, and ED ultrasound screening [10,11,12,13,20,21,22,23,24,25,26]; epidemiology and function in older adults [1,2,3,27,28,29,30]; and narrative/programmatic evidence in colorectal oncology [14,15,31,32,33].

Distribution by domain (overlaps allowed). ERAS standards 2/34 ([5,6]); comparative effectiveness 4/34 ([4,7,8,15]); ED-feasible pre-optimization 9/34 ([9,14,16,17,18,19,31,32,33]); risk stratification 12/34 ([10,11,12,13,20,21,22,23,24,25,26,30]); oncology emergencies 2/34 ([14,15]); epidemiology/system context 7/34 ([1,2,3,27,28,29,30]). The asymmetry—few primary ERAS standards versus a larger body on risk—indicates that some recommendations are anchored more in consensus and feasibility than in multiple randomized comparisons; we therefore grade practice statements as pragmatic and conditional, emphasizing local audit and iterative refinement.

**Figure 2:**
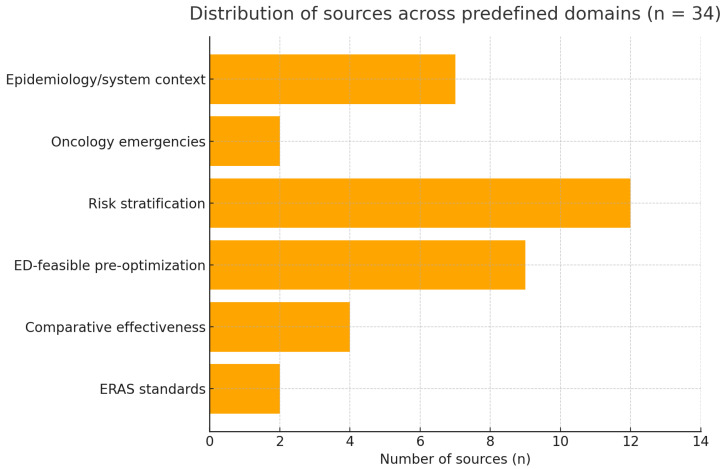
Distribution of sources across predefined domains (n = 34).

Totals exceed 34 due to multi-domain contributions, as prespecified for this scoping review in Figure 2.

### 3.2. ERAS Standards for EL

The ERAS Society guidelines codify a structured, time-sensitive pathway that includes rapid diagnosis, senior-led assessment, risk stratification, targeted optimization, intraoperative multi-modal strategies, and postoperative early feeding and mobilization [5,6]. These guidelines stress the principle of “no delay”: preoperative interventions should never compromise the time to source control.

### 3.3. Evidence of Effect

A meta-analysis of ERAS in emergency abdominal surgery demonstrated reduced LOS and complications [7], and cohort studies have confirmed feasibility with signals for improved outcomes [8]. A systematic review further identified common interventions (nutrition, early mobilization, multi-modal analgesia, fluid balance) and outcome domains (LOS, complications, mortality, functional recovery) as priorities for adoption and audit [4].

Across comparative studies, the most consistent improvements associated with ERAS-aligned pathways were as follows:

- Shorter length of stay (LOS) (typically 1–3 days)
- Lower overall postoperative complications, with pulmonary complications and postoperative ileus reduced in several cohorts, and readmission’s reduced in colorectal ERAS programmes; mortality effects were heterogeneous or neutral across studies [4,7,8,17].

Across comparative studies, the outcomes most consistently improved under ERAS-aligned emergency pathways were: shorter length of stay (typically 1–3 days) and lower overall postoperative complications; several cohorts showed reductions in pulmonary complications and postoperative ileus, and ERAS-colorectal programmes reported fewer re-admissions. Effects on mortality were heterogeneous or neutral across studies [4,7,8,25].

### 3.4. Pre-Optimization Is Feasible in ED

**Figure 3:**
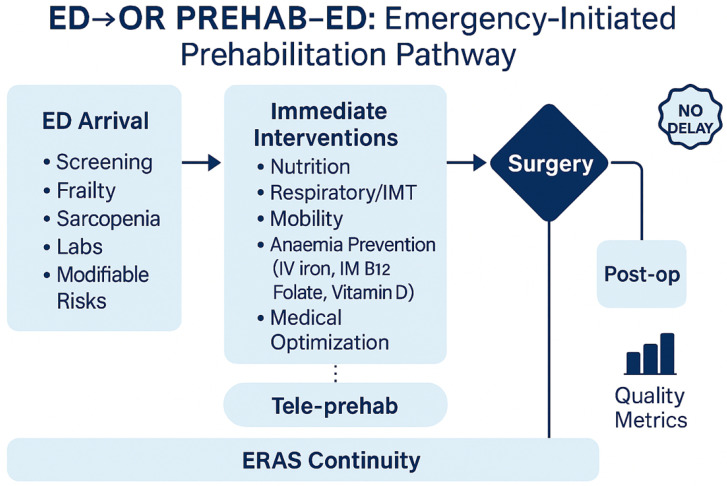
Conceptual framework of an emergency-initiated prehabilitation pathway. At ED arrival, patients undergo rapid screening (frailty, sarcopenia, labs, modifiable risks). Immediate interventions include nutrition, respiratory training, mobility, anemia prevention (IV iron, IM B12, folate, vitamin D), and general medical optimisation. All actions operate under the principle of no delay and must be transferred to the operating room and postoperative care. ERAS continuity and tele-prehab strategies support adherence, with outcomes assessed via quality metrics.

Best-practice guidance supports pragmatic preoperative interventions that can be safely initiated without delay: multi-modal analgesia and goal-directed fluids, early oral supplements if the risk of aspiration is low, respiratory preparation (incentive spirometer or brief inspiratory muscle training), and treatment of reversible comorbidities such as anemia [9]. The 2017 international consensus on peri-operative anemia supports early screening and IV iron in urgent pathways where logistics permit [16]. These actions represent an opportunity to “buy back” physiological reserves during ED waits for diagnostics, OR slots, or senior review (Figure 3).

### 3.5. Targeting High-Risk Patients

Sarcopenia is a strong predictor of adverse outcomes after EL [10], and acute sarcopenia has been described peri-operatively [11]. Frailty similarly increases mortality and complications [12,20]. The ESS offers a rapid, validated risk stratification tool for triage and bundle activation in the ED [13]. ED-based ultrasound of the rectus femoris muscle has been piloted as a feasible biomarker of sarcopenia, providing an immediate bedside measure [21].

### 3.6. Oncology Emergencies and Local Programs

In obstructive colorectal cancer, ERAS-consistent measures are broadly applicable but must be tailored to aspiration risk, obstruction physiology, and sepsis. Narrative evidence supports the feasibility of ERAS in this setting [14]. Contemporary prehabilitation programs demonstrate the feasibility of morphofunctional assessment and combined interventions (oral supplements + exercise) [31], report cost savings from integrated prehabilitation [32], and show improved postoperative outcomes after structured optimization [33].

Implementation, safety, and resource use across the included literature suggest that a minimal ED-initiated bundle is feasible in real-world pathways. Comparative and programmatic reports describe reductions in complications, length of stay, and re-admissions with ERAS-concordant care, acknowledging heterogeneity and context dependence [7,8]. Cost analyses indicate potential savings when prehabilitation elements are integrated into peri-operative care, supporting sustainability arguments for hospital leadership [32]. Program evaluations highlight improved postoperative recovery when optimization is standardized and handed over reliably to OR and ward teams [33]. At the system level, population-based and multi-center cohorts underscore the persistent burden and variability of emergency general surgery outcomes, particularly in older adults, reinforcing the value of structured pathways and audit [1,2,20,27]. Very elderly patients (including nonagenarians) exhibit marked functional decline after emergency surgery, which strengthens the case for prioritized supportive measures and early escalation [28]. In low- and middle-income settings, baseline risk is higher and resources more constrained, emphasizing the need for low-cost, high-yield components and clear “no-delay” governance [3]. Collectively, these signals justify the focus on pragmatic, transferable actions and concise metrics aligned with ERAS [4,5,6].

### 3.7. Advanced Risk Stratification and Emerging Predictors

Recent studies expand risk profiling for emergency laparotomy (EL) beyond traditional clinical scores. A multi-center cohort incorporating CT-based body composition found that both sarcopenia and myosteatosis independently predicted 30-day and 1-year mortality, and improved model discrimination when added to standard clinical variables (AUC 0.84), reinforcing the value of morphometric phenotyping in the emergency setting [22]. In parallel, a systematic review of preoperative EL risk tools (P-POSSUM, POSSUM, NELA, MPI, among others) synthesized 82 observational studies and reported generally accurate mortality prediction across tools, without clear superiority of any single model, supporting local adoption based on familiarity and workflow integration [25]. Beyond scores, a pilot study suggests preoperative immune biomarkers (e.g., suPAR, IL-6, TNF-α) add predictive signal for major complications and mortality when combined with baseline risk, highlighting a pragmatic route to biomarker-augmented triage in the ED [24]. Finally, validated delirium risk algorithms applicable preoperatively (e.g., PIPRA) achieved (AUC 0.8) and include laparotomy as a procedure-level factor; these tools can prioritize preventive bundles for older adults at high delirium risk [26,34].

## 4. Discussion

### 4.1. Principal Findings and Interpretation

This scoping review synthesizes guideline recommendations, comparative evidence, and best-practice reviews to propose a practical, emergency department-initiated, enhanced recovery after surgery (ERAS)-aligned strategy for emergency laparotomy (EL). Across sources, three themes consistently emerge. First, ERAS standards for EL provide a coherent framework spanning rapid assessment and optimization, intraoperative management, and postoperative rehabilitation, with the explicit principle of “no delay” to source control [5,6]. Second, comparative data—though heterogeneous—generally favor ERAS-aligned pathways in emergency abdominal surgery, showing signals for reduced complications and a shorter length of stay (LOS) versus conventional care [7,8]. Third, risk targeting is essential: sarcopenia and frailty are prevalent and strongly predictive of adverse outcomes in EL populations, while pragmatic tools such as the Emergency Surgery Score (ESS) support rapid triage and resource allocation in pressured ED settings [10,11,12,13,14]. Together, these strands justify a minimal, time-efficient pre-optimization bundle that is initiated in the ED, explicitly does not delay the OR, and is seamlessly handed over to intra- and postoperative care.

### 4.2. Why an ED-Initiated Bundle Matters

Systems-level work highlights high utilization and mortality after emergency general surgery (EGS), with wide variation by center—particularly in older adults [1,2]. Contemporary epidemiology points to a substantial, persistent burden of EGS admissions and operations arising directly from the ED [20,27,28], including very elderly patients who experience steep functional decline after surgery [28]. In this context, hours spent in the Emergency Department (ED) or in the acute surgical unit awaiting diagnostics, senior review, or the Operating Room (OR) represent a window to initiate small, safe, physiologically meaningful actions. The ERAS Part 1 guidance explicitly endorses rapid, structured preoperative optimization without postponing surgery [5], while Part 2 emphasizes the continuity and accountability required to carry ED measures forward into the operating room and ward [6]. This “ED-to-OR handover” concept is the keystone that converts opportunistic pre-optimization into an integrated pathway.

### 4.3. Mechanistic Rationale for the Bundle

Four domains are consistently supported across the mapped literature:

1. Analgesia and fluids. Goal-directed fluid therapy and multimodal, opioid-sparing analgesia are core ERAS elements that attenuate physiological stress and reduce pulmonary and ileus-related complications [5,6,7,8]. In emergencies, the aim is not elaborate prehabilitation but fast, protocolized resuscitation and pain control that stabilize physiology without delaying source control [5,9].
2. Nutrition. Early, safe nutrition is linked to preserved lean mass and immune function in elective ERAS. In emergencies, oral nutritional supplements (ONS) can be initiated when the aspiration risk is acceptable and the obstruction physiology allows, with continuation postoperatively [5,9]. Recent program experience supports the feasibility of combined nutrition-exercise interventions and their potential to improve perioperative readiness [31] and generate cost savings [32].
3. Respiratory preparation. Incentive spirometer and brief inspiratory muscle training (IMT), when feasible, are low-risk and may reduce atelectasis-related complications, particularly in high-risk or painful upper-abdominal presentations [9]. Given the compressed timelines, even short exposure alongside coaching can be justified if it does not interfere with OR timing.
4. Pragmatic comorbidity optimization (patient blood management, PBM). Anemia and iron deficiency are common and are associated with transfusions and adverse outcomes. The international consensus advocates early screening and intravenous iron (IVI) when timelines and logistics permit [29]. In emergency pathways, a single-dose IVI strategy during ED/ward wait can be considered for iron-deficiency anemia when it will not delay OR. Where feasible and indicated, targeted correction of vitamin B12, folate, and vitamin D can be incorporated, recognizing that the evidence is extrapolated and the timing is short [29]. The unifying principle is opportunism without delay.

### 4.4. Targeting: Who Stands to Benefit Most

Sarcopenia is consistently associated with complications, prolonged LOS, and mortality after EL [10]. “Acute sarcopenia”—rapid, perioperative loss of muscle mass and function—further jeopardizes recovery trajectories [11]. A Frailty meta-analysis in emergency surgery reinforces higher mortality and complications along the frailty spectrum [12,13,14]. Operationalizing this in the ED requires tools that are fast and actionable. ESS provides a global risk estimate calibrated for EGS and is practical for triage and escalation decisions (e.g., senior review, HDU/ICU liaison, early physiotherapy, and dietetics) [14]. As a complementary approach, point-of-care ultrasound of the rectus femoris offers a feasible morphofunctional marker of sarcopenia in the ED and may help prioritize rehabilitation resources, though further validation for outcome prediction and change sensitivity is needed [21]. In very elderly cohorts—including nonagenarians—functional decline after EGS is striking [28], making proactive, risk-targeted supportive care ethically and clinically compelling.

### 4.5. Integrating Morphometric and Biomarker Data into ED Triage

CT-derived sarcopenia and myosteatosis are reproducible, add independent signal to 30-day and one-year mortality, and improve discrimination of existing models in EL; when a recent CT is available, embedding automated L3 muscle/attenuation reads into the ED→OR pathway could refine resource allocation (ICU liaison, respiratory therapy, nutrition) [22]. Complementarily, immune biomarkers (suPAR, IL-6, TNF-α) measured preoperatively may enhance early prognostication and could be piloted where laboratory turnaround is rapid and does not delay source control [24].

### 4.6. Frailty, Discharge Planning and End-of-Life Implications

The UK-wide ELF cohort (n = 934) demonstrated that preadmission frailty strongly predicts increased care level at discharge, with greater predictive power than age; embedding frailty scoring informs shared decision-making and anticipatory discharge planning [23] Among older EGS decedents, frailty correlates with more intensive end-of-life care, lower hospice use, more ED revisits and ICU admissions in the last 30 days—supporting early palliative involvement and goal-concordant care discussions in high-risk flags [30].

### 4.7. Context: Oncological Emergencies

Obstructive or complicated colorectal cancer (CRC) constitutes a frequent, high-risk EL indication. Narrative evidence indicates that ERAS principles remain applicable if aspiration risk, obstruction physiology, and septic control are respected [16]. These patients may be especially suitable for a short-course, multimodal ED bundle (e.g., ONS if safe, respiratory coaching, mobilization intent, anemia screening) coupled with explicit transfer of orders and early ICU planning where indicated. Contemporary ERAS-colorectal experience suggests that pathway adherence can reduce LOS and readmissions [15]; local programmatic reports describe feasibility, economic benefits, and improved postoperative outcomes after structured optimization/prehabilitation [32,33].

### 4.8. Operative Conduct and Team Configuration

Emergency pathways benefit from dual consultant-led care for high-risk cases, shortened operative duration where feasible, and early involvement of specialized teams (e.g., stoma care), which may mitigate complications and improve recovery. These perioperative levers complement ED-initiated optimization and should be explicitly built into handover checklists [17].

### 4.9. Implementation: From Concept to Routine Practice

Heterogeneity in outcomes across centers [2] and across income settings [3] underscores the need for implementation science. Three enablers recur, as follows:

- Triggers in the EHR. Embedding ESS and a brief sarcopenia/frailty screen as ED prompts can automatically trigger order sets (ONS when safe, incentive spirometer, mobilization intent, CBC/ferritin/TSAT) and a “no-delay” rule [5,6,14].
- Ready-to-use kits and roles. Respiratory devices at triage bays, ONS stock, patient leaflets, and a one-page protocol reduce friction. Role clarity (ED nursing, physiotherapy, dietetics, anesthesia, surgery) mitigates “everyone/no-one” ownership problems and aligns with ERAS Part 2 accountability [6,9].
- Handover and escalation. A templated “ED → OR → ward” handover (checklist plus named owner) preserves fidelity. For high-risk flags (ESS high, severe frailty/sarcopenia), pre-defined escalation (senior surgical review, anesthesia/ICU consult) shortens the time to definitive decisions [6,14].

### 4.10. Implementation Roadmap (Tiered)

We propose progressive adoption that aligns with resource realities:

- Bronze (Minimal): one-page protocol; ESS/CFS triage prompt; stock of ONS and incentive spirometers; analgesia/fluids order set with no-delay hard stop; basic CBC/ferritin/TSAT; named owner for ED → OR handover.
- Silver (Standard): on-call grid for physio/dietitian (response ≥ 2 h); micro-learning modules (20 min); shared ultrasound access; weekly dashboard of process metrics.
- Gold (Enhanced): automated CT-morphometrics; bedside rectus femoris ultrasound with competency log; single-dose IV iron pathway; embedded delirium-risk tool (PIPRA); monthly review of morbidity, costs, and equity.

A structured summary of roles, training, equipment, IT, quality assurance, cost levers, and change-management tactics is provided (Table 1).

### 4.11. Operational Risks and Safeguards

ED crowding and competing priorities can create bottlenecks if prehabilitation tasks are applied rigidly. We embed (i) an eligibility gate tied to ESS/CFS and time-to-OR, (ii) task triage (e.g., nutrition and respiratory actions only when aspiration risk and transport logistics permit), and (iii) abandon/hand-over triggers if theater timing advances or the clinical picture changes. Sites should track time-to-OR and door-to-antibiotics to ensure neutrality or improvement.

### 4.12. Contextual Adaptation

- Rural/remote: Use the Bronze bundle; paper checklist; shared ultrasound if available or no-tech proxies (hand-grip, calf circumference); tele-physio and tele-dietitian; ONS trolley in ED.
- Resource-constrained/LMIC: Prioritize time-neutral actions (analgesia/fluids, early mobilization intent, delirium prevention); substitute IV iron with early post-op oral iron + folate when IV supply/logistics limit delivery; use CFS when ESS/EHR tools are unavailable; monitor two core metrics (time-to-OR, LOS).
- Different organizational structures: If anesthesia leads pre-operative assessment, ED nursing initiates and transfers the bundle via a preformatted handover; if pharmacy controls stock, create ED kits (ONS, spirometers).

### 4.13. Measurement and Learning

Adoption benefits from concise metrics that are easy to capture and meaningful for both teams and managers. Process indicators include: completion rates of ESS and sarcopenia/frailty screens; time to antibiotics and to the operating room; initiation of ONS/respiratory coaching when eligible; and documentation of handover orders. Early clinical outcomes include pulmonary complications, delirium proxies, LOS, discharge destination, transfusion exposure, and 30-day readmission; functional outcome tracking is desirable where feasible [4,6,7,8,31,33]. Regular audit-feedback cycles can progressively reduce variation and strengthen adherence, addressing the center-to-center variability documented in EGS [2]. Program reports demonstrating cost savings from prehabilitation can be leveraged to sustain institutional support [32].

### 4.14. Evaluation Framework (For Research and QI)

A core set enables audits and pragmatic trials:

- Process: completion rates of ESS/CFS; ONS started when eligible; respiratory coaching delivered; ED → OR handover completed; no-delay breaches.
- Clinical (30-day): pulmonary complications, postoperative ileus, delirium proxy, transfusion exposure, length of stay, discharge destination, readmissions, mortality.
- Implementation: fidelity (bundle adherence %), acceptability (staff survey), cost (pharmacy/physio time, ONS/IV iron), and equity (age/frailty strata).

Data capture points: ED departure, post-op day 3, discharge; linkage to routine EHR metrics is encouraged.

### 4.15. Equity and Context Sensitivity

Outcomes after EGS are particularly vulnerable to resource constraints, with LMIC settings facing structural barriers to guideline implementation [3]. The proposed ED-initiated bundle is intentionally minimal, low-cost, and designed to fit varied contexts: it prioritizes screening and supportive actions that are quick, safe, and likely to yield benefits even when specialist staffing is limited. Point-of-care approaches (e.g., rectus femoris ultrasound) may democratize risk assessment where CT-based body composition is unavailable [21]. Nevertheless, successful implementation in constrained settings requires local adaptation, phased roll-out, and explicit attention to supply chains (e.g., ONS, IV iron availability).

### 4.16. Safety Considerations and “No-Delay” Governance

The single most important guardrail is that pre-optimization must never delay source control. All elements in the bundle are optional and opportunistic; they should be aborted or deferred at the first hint of interference with time-to-OR targets. Nutrition is initiated only when the aspiration risk is low; respiratory training must not impede necessary analgesia, imaging, or transport; PBM actions (e.g., IV iron) are pursued only when they are logistically neutral to OR timing [5,6,9,29].

The 2022 ESC Guideline for non-cardiac surgery provides practice-ready recommendations on cardiovascular risk assessment, (re)starting statins, handling antithrombotics/anticoagulants, and peri-operative hemodynamic strategies; aligning our ED bundle with these guidance points improves safety without compromising timing to OR [18]. In addition, perioperative dyslipidemia has been associated with higher postoperative morbidity/mortality and may alter anesthetic pharmacokinetics; while emergency timelines preclude full lipid optimization, flagging uncontrolled profiles for early post-op management is reasonable [19].

Embedding the “no-delay” rule into the EHR order set reduces the risk of mission creep.

### 4.17. How This Review Advances the Field

Compared with existing guidance and reviews, our synthesis (i) integrates ED-specific feasibility with ERAS standards, (ii) explicitly prioritizes risk-targeted deployment using ESS and sarcopenia/frailty, and (iii) translates the evidence into a minimal, handover-anchored bundle suitable for immediate adoption. By situating pre-optimization in the ED and defining practical enablers and metrics, the review bridges the gap between elective prehabilitation paradigms and the realities of urgent care.

### 4.18. Limitations of the Evidence Base and of This Review

Evidence supporting ERAS in emergencies is heterogeneous and often observational, with variability in pathway composition and fidelity [7,8]. Randomized evidence for individual pre-optimization components in true emergency timelines is scarce; PBM recommendations are consensus-based and largely extrapolated from elective pathways [29]. The prognostic role of sarcopenia/frailty is clear, but the demonstration of reversibility within the limited ED-to-OR window remains limited [10,11,12,13]. As a scoping review, our synthesis privileges breadth and transferability over pooled effect sizes and does not perform risk-of-bias grading. These limitations reinforce the need for pragmatic implementation research.

### 4.19. Practice Recommendations

We recommend the following:

1. Activate a minimal, ED-initiated, ERAS-aligned bundle comprising multi-modal analgesia, goal-directed fluids, early safe nutrition, respiratory preparation, mobilization intent, and pragmatic PBM (CBC, ferritin/TSAT; consider IV iron when indicated/logistically neutral; correct B12/folate/vitamin D as appropriate) under an explicit no-delay rule [5,6,9,16].
2. Target deployment using ESS and a brief sarcopenia/frailty screen; consider rectus femoris ultrasound to refine prioritization where feasible [10,11,12,13,21].
3. Embed EHR triggers, ready-to-use kits, and named ownership for ED → OR → ward handover; pre-define escalation for high-risk flags [6,13].
4. Monitor a concise metric set and feed results back to teams; leverage economic and outcome gains from local programs to maintain support [4,7,8,15,32,33].

### 4.20. Research Agenda

Priorities include the following: cluster-pragmatic evaluations of the ED bundle versus usual care; fidelity, cost, and equity reporting; core outcome sets aligned with emergency ERAS audits; operational validation of ED rectus femoris ultrasound as a risk and response biomarker; implementation of urgent PBM algorithms with time-to-OR neutrality; and dedicated evaluations in oncological emergencies, especially obstructive CRC [4,6,14,15,21,31,32,33]. Studies should deliberately include very elderly and frail cohorts given their disproportionate risk [12,20,27,28,29].

### 4.21. Ethical Considerations

Non-urgent components (e.g., ONS, incentive spirometry) require verbal consent when capacity is present. For reduced capacity, we apply local surrogate/Best-Interest frameworks and document time neutrality. IV iron or IM vitamin B12 should be discussed when feasible; otherwise, defer to early post-operative care. Patient autonomy is preserved by opt-out without affecting core resuscitation. Allocation uses transparent criteria (ESS/CFS risk) to balance individual benefit and system efficiency.

## 5. Conclusions

A targeted, ED-initiated, no-delay bundle that is explicitly integrated with ERAS intra- and postoperative care is biologically plausible, guideline-concordant, implementable, and supported by favorable signals in the emergency literature. By aligning risk stratification with pragmatic pre-optimization and reliable handover, centers can reasonably expect to reduce complications and LOS while addressing unwarranted variation in EL outcomes. 

## Figures and Tables

**Table 1:** Implementation roadmap for ED-initiated prehabilitation (tiered).

Domain	Bronze (Minimal)	Silver (Standard)	Gold (Enhanced)
Staffing & roles	ED nurse triggers checklist; surgeon/ED physician co-own; basic physio/dietitian on-call	Named “bundle owner” per shift; physio/dietitian response ≤2 h	Perioperative team huddle; dual consultant availability for high-risk
Training	20 min micro-learning; one-page protocol	Annual refresh; IMT coaching script	RF ultrasound competency log; CT-morphometrics auto-feed
Equipment/supplies	ONS stock; incentive spirometers; printed checklists	Shared ultrasound; single-dose IV iron kit; stoma nurse pager	Point-of-care RF-US; spirometry counters; digital leaflets
IT/EHR	ESS/CFS prompt; order set with no-delay hard stop	Auto handover ED → OR; weekly process dashboard	CT auto-read (L3 area/attenuation); PIPRA integration
Quality assurance	Track time-to-OR, screening rates	Add LOS, pulmonary events, ileus, readmission	Add fidelity %, cost, staff acceptability, equity
Cost levers	Minimal (stock + training)	Pharmacy kits + part-time physio/dietitian	Ultrasound devices; analytics support
Change management	Local champion; brief huddles	Audit–feedback monthly	Formal QI cycles; cross-site learning

## Supplementary Materials

The following supporting information can be downloaded at https://www.mdpi.com/article/10.3390/jcm14196922/s1, Supplementary Material S1: PRISMA-ScR Checklist; Table S1: Characteristics of included studies in the scoping review; Table S2: Methodological Appraisal Signals by Study.

## Abbreviations

The following abbreviations are used in this manuscript:

CFS	Clinical Frailty Scale
EL	Emergency Laparotomy
ERAS	Enhanced Recovery After Surgery
ESS	Emergency Surgery Score
IMT	Inspiratory muscle training
ONS	Oral Nutritional Supplements
PBM	Patient Blood Management
PIPRA	Pre-Interventional Preventive Risk Assessment
RF-US	Rectus Femoris ultrasound
suPAR	Soluble Urokinase Plasminogen Activator Receptor
