# Supplementary material for: From Emergency Department to Operating Room: The Role of Early Prehabilitation and Perioperative Care in Emergency Laparotomy: A Scoping Review and Practical Proposal
Source: J Clin Med. 2025 Sep 30;14(19):6922. doi: 10.3390/jcm14196922 (PMC12525299; doi:10.3390/jcm14196922)
Supplement: Supplementary file 1 [file jcm-14-06922-s001.zip › Supplementary Material S1.pdf]

Supplementary Material S1 – PRISMA-ScR Checklist

We followed the PRISMA Extension for Scoping Reviews (PRISMA-ScR). The completed checklist is provided below.

Section	***Item***	***Description***	***Reported in manuscript***
Title	1	Identify the report as a scoping review	Title page
Abstract	2	Structured summary	Abstract
Rationale	3	Rationale for review	Introduction
Objectives	4	Explicit statement of questions	Introduction
Eligibility criteria	5	Inclusion/exclusion (PCC)	Methods
Information sources	6	Databases, search dates	Methods
Search	7	Full search strategy	Methods (summarised)
Selection of sources	8	Process for selecting sources	Methods
Data charting	9	Data charting process	Methods
Data items	10	Variables extracted	Methods
Critical appraisal	11	Not applicable (scoping)	N/A
Synthesis of results	12	Approach to synthesis	Methods
Results	13	Selection of sources (numbers)	Results + Fig S1
Characteristics of sources	14	Study characteristics	Table S2
Results by domain	15	Mapped interventions/outcomes	Results
Discussion	16	Summary of evidence, gaps, implications	Discussion
Limitations	17	Limitations of evidence	Discussion
Conclusions	18	Interpretation and next steps	Conclusion
Funding	19	Sources of funding	Declarations
