# Supplementary material for: From Emergency Department to Operating Room: The Role of Early Prehabilitation and Perioperative Care in Emergency Laparotomy: A Scoping Review and Practical Proposal
Source: J Clin Med. 2025 Sep 30;14(19):6922. doi: 10.3390/jcm14196922 (PMC12525299; doi:10.3390/jcm14196922)
Supplement: Supplementary file 1 [file jcm-14-06922-s001.zip › Supplementary Table S1.pdf]

Supplementary Table S1. Characteristics of included studies in the scoping review.

Caption: Overview of the 34 included sources, grouped by domain (guidelines, comparative effectiveness, ED-feasible pre-optimisation, risk stratification, oncology emergencies, epidemiology/system context). Outcomes and notes emphasise feasibility, signals of effect, and implementation points. Bracketed numbers refer to the reference list [1–34].

First Author (Year)	***Study Type***	***Population/Setting***	***Intervention / Focus***	***Outcomes***	***Key Notes***
Peden (2021) [5]	ERAS Guideline	Adults, EL	Preop standards	Recommendations	ERAS Part 1; “no-delay”
Scott (2023) [6]	ERAS Guideline	Adults, EL	Intra/post-op ERAS	Recommendations	ERAS Part 2; handover/accountability
Harji (2025) [4]	Systematic review	EL pathways	Key interventions/outcomes	Mortality, LOS, complications	Domains to guide adoption/audit
Hajibandeh (2020) [7]	Meta-analysis	Emergency abdominal	ERAS protocols	↓Complications, ↓LOS	Heterogeneity across programs
Wisely (2016) [8]	Cohort	Emergency surgery	ERAS vs standard	LOS, complications	Feasible; positive signal
Poulton (2019) [9]	Best-practice review	EL	Pre-optimisation	N/A	“No-delay” actions; ED-start feasible
Muñoz (2017) [16]	Consensus	Perioperative	Anaemia/PBM (IV iron)	Recommendations	Early screening/IV iron if feasible
Humphry (2023) [10]	Systematic review	EL	Sarcopenia	Mortality, complications	Strong prognostic signal
Shrestha (2022) [11]	Review	Elective & EL	Acute sarcopenia	Functional decline	Perioperative muscle loss concept

Leiner (2022) [12]	Systematic review	EGS	Frailty	Mortality, complications	Frailty meta-analytic risk
Halle-Smith (2021) [20]	Review	Elderly, EGS	Frailty pathways	Functional outcomes	Geriatric-sensitive care
Saxena (2022) [13]	Narrative review	EGS	ESS score	Risk stratification	Practical ED tool
García-Sánchez (2025) [21]	Cross-sectional	ED	Rectus femoris US	Sarcopenia detection	Feasible bedside biomarker
Mihailescu (2025) [14]	Narrative review	Complicated CRC	ERAS oncology	Feasibility	Strict “no-delay” governance
Mudarra (2025) [31]	Feasibility	Surgical candidates	Morphofunctional + ONS/exercise	Adherence, feasibility	Short-course prehab
Mudarra (2025) [32]	Cost analysis	Major surgery	Prehabilitation	Cost savings	Economic benefit
García-Sánchez (2024) [33]	Program evaluation	Surgical pts	Presurgical optimisation	Post-op results	Improved recovery
Shah (2017) [15]	Cohort	Colorectal ERAS	ERAS protocol	↓Readmissions, ↓LOS	Supports ERAS adherence
Lee (2020) [1]	Population cohort	Medicare, EGS	Outcomes vs acute medicine	Mortality, utilisation	System-level burden
Ingraham (2011) [2]	Cohort	Elderly, EGS	Quality of care	Variation	Centre-level variability
Shah (2015) [3]	LMIC cohort	Elderly, EGS	Outcomes	Mortality	LMIC vulnerability

Harada (2024) [29]	Cohort	≥90 yrs, EGS	Post-op function	Performance decline	Very elderly trajectories
Park (2023) [28]	Cohort	Elderly EGS	Trends	LOS, mortality	Rising demand
Fehlmann (2024) [27]	Retrospective	ED→EGS	Incidence & outcomes	Mortality, utilisation	High ED-to-OR burden
Leinner (2022) [12]	Sys. review	EGS	Frailty→mortality	Mortality	Confirms frailty risk
Body (2022) [22]	Multicentre cohort	EL	CT sarcopenia & myosteatosis	30-day & 1-yr mortality	Morphometrics add discrimination
Carter (2020) [23]	Cohort (ELF)	EL	Preadmission frailty	Care level at discharge	Frailty > age for disposition
P. Hasselager (2021) [24]	Pilot cohort	EL	Immune biomarkers (suPAR, IL-6)	Major comps, mortality	Biomarker-augmented risk
Hewitt (2024) [25]	Sys. review	EL	Risk tool performance	Discrimination/calibration	NELA, POSSUM, etc.; none dominant
Sokas (2021) [30]	Cohort	Older EGS	Frailty & EOL intensity	ICU days, hospice use	Early palliative triggers
Boyd-Carson (2020) [17]	Review	EL	Surgical/peri-op factors	N/A	Dual consultant, stoma care, timing
Halvorsen (2022) [18]	Guideline	Non-cardiac surgery	CV assessment/management	Recommendations	Statins, antithrombotics, hemodynamics
Ramirez-Reyes (2021) [19]	Sys. review	Perioperative	Dyslipidaemia & outcomes	Complications	Flag for early post-op mgmt

Dodsworth (2023) [26]	Model development/validation	Surgical	PIPRA delirium prediction	AUC ~0.8	Preop delirium risk tool (CE-marked)

Abbreviations: EL = emergency laparotomy; EGS = emergency general surgery; ERAS = Enhanced Recovery After Surgery; LOS = length of stay; PBM = patient blood management; CRC = colorectal cancer; ONS = oral nutritional supplements; US = ultrasound; EOL = end-of-life.
