# Supplementary material for: From Emergency Department to Operating Room: The Role of Early Prehabilitation and Perioperative Care in Emergency Laparotomy: A Scoping Review and Practical Proposal
Source: J Clin Med. 2025 Sep 30;14(19):6922. doi: 10.3390/jcm14196922 (PMC12525299; doi:10.3390/jcm14196922)
Supplement: Supplementary file 1 [file jcm-14-06922-s001.zip › Supplementary_Table S2.pdf]

Supplementary Table S2. Methodological Appraisal Signals by Study

Author, Year	Design / Context	Domain	Methodological Appraisal Signals
Hajibandeh, 2020	Systematic review of 14 studies in EL	Comparative effectiveness	Moderate heterogeneity, limited RCT evidence, but useful synthesis of available data.
Peden, 2021	ERAS Society multinational guideline for EL	ERAS standards	High face validity, consensus-driven, lacks direct empirical validation.
Poulton, 2019	Narrative review on pre-optimisation in EL	ED-feasible pre-optimization	Theoretical feasibility, no primary data, highlights key gaps.
Wisely, 2016	Retrospective cohort, Australia, n=250	Epidemiology/system context	Single-centre, observational; shows improved LOS; risk of selection bias.
Carter, 2020	Cohort, UK, EL patients	Risk stratification	Identified sarcopenia and frailty as key predictors; limited external validity.
Boyd-Carson, 2020	Review on perioperative factors in EL	Epidemiology/system context	Broad scope; narrative synthesis; lacks standardized methodology.
Muñoz, 2016	International consensus statement on perioperative anaemia	Anaemia/Comorbidity Optimization	Strong consensus, multidisciplinary input; not trial-based.
Lee, 2019	Cohort, USA, EL patients	Risk stratification	Large sample, clear outcomes; observational design limits causality.
Petring Hasselager, 2020	Pilot study on mortality and major complications	Comparative effectiveness	Small sample size; pilot design limits generalizability.
Shah, 2017	Cohort, UK, EL	Risk stratification	Validated ESS score;

	patients		good predictive value; retrospective limitations.
World J Surg, 2021 (Peden)	Guideline on perioperative care for EL	ERAS standards	Comprehensive framework; consensus-based; limited empirical testing.
ANZ J Surg, 2016 (Wisely)	Cohort study in Australia	Epidemiology/system context	Enhanced recovery principles feasible; observational nature limits inference.
Cureus, 2023	Observational study	Risk stratification	Highlights frailty impact; small sample; single-centre limitations.
J Med Life, 2025	Review	Epidemiology/system context	Synthesizes emerging evidence; narrative scope limits appraisal strength.
PIPRA validation, 2023	Prospective validation study	Risk stratification	Promising predictive value; requires external validation.
suPAR study, 2020	Biomarker-based cohort	Risk stratification	Novel biomarker; adds predictive capacity; limited widespread availability.
PBM consensus, 2020	Guideline	Anaemia/Comorbidity Optimization	Consensus-based; strong recommendations; limited RCT backing.
Frailty/sarcopenia review, 2022	Narrative review	Nutritional optimization & Sarcopenia	Highlights clinical importance; limited trial evidence.
Nutrients, 2024	Cohort study	Nutritional optimization & Sarcopenia	Explores vitamin D and nutrition; observational nature.
JCM, 2024	Cohort study	Risk stratification	ESS validated; retrospective design; generalizable findings.
Acta Anaesthesiol Scand, 2020	Cohort pilot study	Comparative effectiveness	Small pilot; feasibility tested; limited statistical power.
Swiss Med Wkly, 2024	Cohort, Switzerland	Epidemiology/system context	Population-level data; robust design; generalizable.
Front Med, 2022	Review	Nutritional optimization & Sarcopenia	Explores multimodal prehabilitation; mostly elective data extrapolated.

Scand J Trauma, 2020	Observational	Epidemiology/system context	Large dataset; heterogeneous populations; lacks RCT support.
Rev Med And, 2023	Cohort, Spain	Risk stratification	Explores comorbidity indices; single-country design.
Cirugía no cardiaca, 2023	Review	Anaemia/Comorbidity Optimization	Spanish guideline; consensus, limited trials.
Ad, 2023	Observational	Risk stratification	Explores myosteatosis; emerging area; requires validation.
Hajibandeh, 2020b	Systematic review update	Comparative effectiveness	Confirms trends; moderate heterogeneity.
Journal ref, 2021	Prospective cohort	Oncology emergencies	Focus on cancer patients; small sample size.
Guideline ref, 2019	Consensus guideline	Anaemia/Comorbidity Optimization	Expert consensus; limited direct evidence.
Nutrients, 2025	Cohort, nutritional support	Nutritional optimization & Sarcopenia	B12 and folate supplementation explored; observational.
ERAS update, 2024	Review	ERAS standards	Updated recommendations; limited EL-specific validation.
Oncology cohort, 2023	Retrospective cohort	Oncology emergencies	Cancer surgery outcomes; high external validity; retrospective design.
